# (2*E*,4*E*)-1-(6-Chloro-2-methyl-4-phenyl-3-quinol­yl)-5-phenyl­penta-2,4-dien-1-one

**DOI:** 10.1107/S1600536810016429

**Published:** 2010-05-12

**Authors:** Wan-Sin Loh, Hoong-Kun Fun, A. J. Viji, S. Sarveswari, V. Vijayakumar

**Affiliations:** a X-ray Crystallography Unit, School of Physics, Universiti Sains Malaysia, 11800 USM, Penang, Malaysia; b Organic Chemistry Division, School of Advanced Sciences, VIT University, Vellore 632 014, India

## Abstract

In the title compound, C_27_H_20_ClNO, the quinoline ring forms a dihedral angle of 62.53 (5)° with the substituent benzene ring. In the crystal, inter­molecular C—H⋯Cl inter­actions link the mol­ecules into chains along the *b* axis. Inter­molecular C—H⋯N and C—H⋯O hydrogen bonds further consolidate the structure into a three-dimensional network. The unit cell contains four solvent-accessible voids, each with a volume of 35 Å^3^, but no significant electron density was found in them.

## Related literature

For the background to and the biological activity of quinolines, see: Bhat *et al.* (2005 ▶); Markees *et al.* (1970 ▶); Campbell *et al.* (1998 ▶). For related structures, see: Loh *et al.* (2010*a* ▶,*b* ▶); Shahani *et al.* (2010 ▶). For the stability of the temperature controller used for the data collection, see: Cosier & Glazer (1986 ▶).
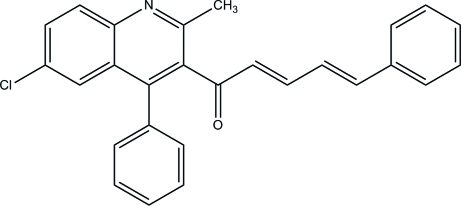


## Experimental

### 

#### Crystal data

C_27_H_20_ClNO
*M* *_r_* = 409.89
Monoclinic, 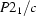

*a* = 6.2464 (3) Å
*b* = 22.5672 (11) Å
*c* = 15.2748 (7) Å
β = 94.620 (1)°
*V* = 2146.20 (18) Å^3^
*Z* = 4
Mo *K*α radiation
μ = 0.20 mm^−1^
*T* = 100 K
0.35 × 0.26 × 0.13 mm

#### Data collection

Bruker APEXII DUO CCD area-detector diffractometer
Absorption correction: multi-scan (*SADABS*; Bruker, 2009 ▶) *T*_min_ = 0.935, *T*_max_ = 0.975
22989 measured reflections
6191 independent reflections
4889 reflections with *I* > 2σ(*I*)
*R*_int_ = 0.033

#### Refinement

*R*[*F* ^2^ > 2σ(*F* ^2^)] = 0.040
*wR*(*F* ^2^) = 0.116
*S* = 1.03
6191 reflections
351 parameters
H atoms treated by a mixture of independent and constrained refinement
Δρ_max_ = 0.38 e Å^−3^
Δρ_min_ = −0.33 e Å^−3^

### 

Data collection: *APEX2* (Bruker, 2009 ▶); cell refinement: *SAINT* (Bruker, 2009 ▶); data reduction: *SAINT*; program(s) used to solve structure: *SHELXTL* (Sheldrick, 2008 ▶); program(s) used to refine structure: *SHELXTL*; molecular graphics: *SHELXTL*; software used to prepare material for publication: *SHELXTL* and *PLATON* (Spek, 2009 ▶).

## Supplementary Material

Crystal structure: contains datablocks global, I. DOI: 10.1107/S1600536810016429/fj2295sup1.cif

Structure factors: contains datablocks I. DOI: 10.1107/S1600536810016429/fj2295Isup2.hkl

Additional supplementary materials:  crystallographic information; 3D view; checkCIF report

## Figures and Tables

**Table 1:** Hydrogen-bond geometry (Å, °)

*D*—H⋯*A*	*D*—H	H⋯*A*	*D*⋯*A*	*D*—H⋯*A*
C3—H3*A*⋯N1^i^	0.966 (17)	2.484 (19)	3.3681 (16)	152.1 (14)
C11—H11*A*⋯Cl1^ii^	0.994 (17)	2.772 (16)	3.6491 (12)	147.4 (14)
C17—H17*A*⋯O1^iii^	1.000 (19)	2.596 (19)	3.4398 (17)	142.1 (14)
C27—H27*C*⋯O1^iv^	0.984 (18)	2.517 (19)	3.3892 (16)	147.6 (14)

Symmetry codes: (i) 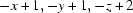; (ii) 
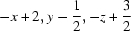
; (iii) 
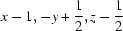
; (iv) 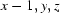.

**Fig. 1.**
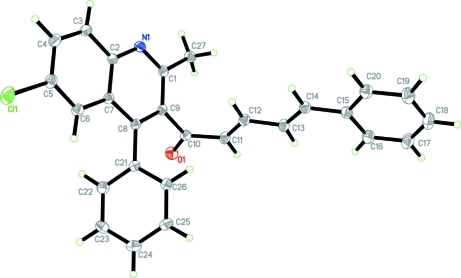
The molecular structure of the title compound, showing 50% probability displacement ellipsoids and the atom-numbering scheme.

**Fig. 2.**
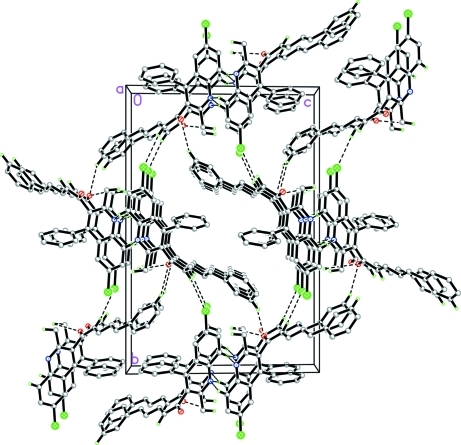
The crystal packing of the title compound, viewed approximately along the a axis, showing the three-dimensional network. H atoms not involved in the intermolecular interactions (dashed lines) have been omitted for clarity.

## supplementary crystallographic information

### Comment

The quinoline derivatives are very important compounds because of their wide occurrence in natural products and biologically active compounds (Markees *et al.*, 1970; Campbell *et al.*, 1998). A variety of natural compounds such as vinblastin, combretastatin A-4 and colchicine attack microtubules by interfering with the dynamics of tubulin polymerization and depolymerization, resulting in mitotic arrest. For a structurally simple group of compounds, chalcones have displayed an impressive array of biological activities, among which anti-malarial, anti-protozoal, anti-inflammatory, immunomodulatory, nitric oxide inhibition, tyronase inhibition, cytotoxic and anticancer activities have been cited in the literature (Bhat *et al.*, 2005). In continuation our interest in synthesis of chalcones herein we report a new chalcone (Loh *et al.*, 2010*a*,*b*; Shahani *et al.*, 2010).

In the title compound (Fig. 1), the quinoline ring system (C1/N1/C2–C9) is approximately planar with a maximum deviation of 0.009 (1) Å at atom C1. This mean plane of quinoline ring system forms a dihedral angle of 62.53 (5)° with the benzene ring (C21–C26) attached to it. Another benzene ring (C15–C20) is linked with the quinoline ring system by a linkage of pentadione (C10–C14/O1) with a dihedral angle of 80.31 (5)° and the torsion angle between the linkage and the quinoline ring system, C8–C9–C10–C11 is 111.08 (13)°. Bond lengths and angles are comparable to closely related structures (Loh *et al.*, 2010*a*,*b*; Shahani *et al.*, 2010).

In the crystal packing (Fig. 2), intermolecular C11—H11A···Cl1 interactions (Table 1) link the molecules into chains down the *b* axis. Intermolecular C3—H3A···N1, C17—H17A···O1 and C27—H27C···O1 hydrogen bonds (Table 1) further consolidate the structure into a three-dimensional network. The unit cell contains four solvent-accessible voids each with a volume of 35 Å^3^. Application of the PLATON SQUEEZE procedure (Spek, 2009) showed no electron count in the void.

### Experimental

A mixture of 3-acetyl-6-chloro-2-methyl-4-phenylquinoline (0.01 M) and cinnamaldehyde (0.01 M) and a catalytic amount of KOH in distilled ethanol was stirred for about 12 h, the resulting mixture was concentrated to remove ethanol then poured on to ice and neutralized with dilluted acetic acid. The resultant solid was filtered, dried and purified by column chromatography using 1:1 mixture of ethyl acetate and petroleum ether. *M.P.*: 416–417 K. Yield: 62 %. The solvent used for the crystallisation was a 1:1 mixture of ethyl acetate and petroleum ether.

### Refinement

All H atoms were located from a difference Fourier map and refined freely [C–H = 0.965 (18) to 1.033 (19) Å].

### Crystal data

**Table:**

C_27_H_20_ClNO	*F*(000) = 856
*M**_r_* = 409.89	*D*_x_ = 1.269 Mg m^−^^3^
Monoclinic, *P*2_1_/*c*	Mo *K*α radiation, λ = 0.71073 Å
Hall symbol: -P 2ybc	Cell parameters from 7467 reflections
*a* = 6.2464 (3) Å	θ = 2.7–30.1°
*b* = 22.5672 (11) Å	µ = 0.20 mm^−^^1^
*c* = 15.2748 (7) Å	*T* = 100 K
β = 94.620 (1)°	Block, yellow
*V* = 2146.20 (18) Å^3^	0.35 × 0.26 × 0.13 mm
*Z* = 4	

### Data collection

**Table:**

Bruker APEXII DUO CCD area-detector diffractometer	6191 independent reflections
Radiation source: fine-focus sealed tube	4889 reflections with *I* > 2σ(*I*)
graphite	*R*_int_ = 0.033
φ and ω scans	θ_max_ = 30.0°, θ_min_ = 1.6°
Absorption correction: multi-scan (*SADABS*; Bruker, 2009)	*h* = −8→8
*T*_min_ = 0.935, *T*_max_ = 0.975	*k* = −31→30
22989 measured reflections	*l* = −21→21

### Refinement

**Table:**

Refinement on *F*^2^	Primary atom site location: structure-invariant direct methods
Least-squares matrix: full	Secondary atom site location: difference Fourier map
*R*[*F*^2^ > 2σ(*F*^2^)] = 0.040	Hydrogen site location: inferred from neighbouring sites
*wR*(*F*^2^) = 0.116	H atoms treated by a mixture of independent and constrained refinement
*S* = 1.03	*w* = 1/[σ^2^(*F*_o_^2^) + (0.0584*P*)^2^ + 0.7188*P*] where *P* = (*F*_o_^2^ + 2*F*_c_^2^)/3
6191 reflections	(Δ/σ)_max_ < 0.001
351 parameters	Δρ_max_ = 0.38 e Å^−^^3^
0 restraints	Δρ_min_ = −0.32 e Å^−^^3^

### Special details

**Table:**

Experimental. The crystal was placed in the cold stream of an Oxford Cryosystems Cobra open-flow nitrogen cryostat (Cosier & Glazer, 1986) operating at 100.0 (1) K.
Geometry. All esds (except the esd in the dihedral angle between two l.s. planes) are estimated using the full covariance matrix. The cell esds are taken into account individually in the estimation of esds in distances, angles and torsion angles; correlations between esds in cell parameters are only used when they are defined by crystal symmetry. An approximate (isotropic) treatment of cell esds is used for estimating esds involving l.s. planes.
Refinement. Refinement of *F*^2^ against ALL reflections. The weighted *R*-factor wR and goodness of fit *S* are based on *F*^2^, conventional *R*-factors *R* are based on *F*, with *F* set to zero for negative *F*^2^. The threshold expression of *F*^2^ > σ(*F*^2^) is used only for calculating *R*-factors(gt) etc. and is not relevant to the choice of reflections for refinement. *R*-factors based on *F*^2^ are statistically about twice as large as those based on *F*, and *R*- factors based on ALL data will be even larger.

### Fractional atomic coordinates and isotropic or equivalent isotropic displacement parameters (Å^2^)

**Table:**

	*x*	*y*	*z*	*U*_iso_*/*U*_eq_	
Cl1	0.98899 (7)	0.715737 (13)	0.90927 (2)	0.02946 (10)	
O1	1.25735 (15)	0.37767 (4)	0.78500 (6)	0.0222 (2)	
N1	0.74535 (17)	0.46489 (5)	0.93300 (6)	0.0166 (2)	
C1	0.8209 (2)	0.42457 (5)	0.88123 (7)	0.0157 (2)	
C2	0.8083 (2)	0.52252 (5)	0.92445 (7)	0.0150 (2)	
C3	0.7221 (2)	0.56504 (6)	0.98060 (8)	0.0184 (2)	
C4	0.7763 (2)	0.62357 (6)	0.97496 (8)	0.0201 (2)	
C5	0.9190 (2)	0.64118 (5)	0.91295 (8)	0.0198 (3)	
C6	1.0057 (2)	0.60204 (5)	0.85712 (7)	0.0179 (2)	
C7	0.9518 (2)	0.54095 (5)	0.86241 (7)	0.0148 (2)	
C8	1.03265 (19)	0.49667 (5)	0.80677 (7)	0.0141 (2)	
C9	0.9673 (2)	0.43886 (5)	0.81688 (7)	0.0146 (2)	
C10	1.0682 (2)	0.39015 (5)	0.76648 (7)	0.0165 (2)	
C11	0.9424 (2)	0.35894 (5)	0.69584 (8)	0.0196 (2)	
C12	0.7385 (2)	0.37191 (5)	0.66730 (8)	0.0186 (2)	
C13	0.6247 (2)	0.34261 (6)	0.59338 (8)	0.0199 (2)	
C14	0.4235 (2)	0.35703 (5)	0.56358 (8)	0.0183 (2)	
C15	0.2997 (2)	0.33203 (5)	0.48666 (7)	0.0177 (2)	
C16	0.3789 (2)	0.28695 (6)	0.43480 (8)	0.0222 (3)	
C17	0.2553 (3)	0.26556 (6)	0.36195 (9)	0.0266 (3)	
C18	0.0520 (3)	0.28854 (6)	0.33945 (9)	0.0271 (3)	
C19	−0.0284 (2)	0.33317 (6)	0.38964 (9)	0.0258 (3)	
C20	0.0949 (2)	0.35471 (6)	0.46291 (8)	0.0216 (3)	
C21	1.1795 (2)	0.51281 (5)	0.73818 (7)	0.0150 (2)	
C22	1.3818 (2)	0.53707 (6)	0.76042 (8)	0.0202 (2)	
C23	1.5169 (2)	0.55152 (6)	0.69544 (9)	0.0224 (3)	
C24	1.4518 (2)	0.54137 (6)	0.60747 (8)	0.0218 (3)	
C25	1.2500 (2)	0.51754 (6)	0.58481 (8)	0.0198 (2)	
C26	1.1141 (2)	0.50339 (5)	0.64940 (7)	0.0169 (2)	
C27	0.7482 (2)	0.36175 (6)	0.89417 (8)	0.0198 (2)	
H3A	0.623 (3)	0.5505 (7)	1.0210 (10)	0.022 (4)*	
H4A	0.715 (3)	0.6527 (8)	1.0137 (11)	0.028 (4)*	
H6A	1.105 (3)	0.6164 (7)	0.8149 (11)	0.024 (4)*	
H11A	1.022 (3)	0.3273 (7)	0.6669 (11)	0.027 (4)*	
H12A	0.658 (3)	0.4025 (7)	0.6958 (11)	0.023 (4)*	
H13A	0.705 (3)	0.3126 (8)	0.5649 (11)	0.029 (4)*	
H14A	0.352 (2)	0.3879 (7)	0.5961 (10)	0.018 (4)*	
H16A	0.527 (3)	0.2710 (8)	0.4492 (12)	0.031 (4)*	
H17A	0.316 (3)	0.2329 (8)	0.3271 (12)	0.033 (5)*	
H18A	−0.032 (3)	0.2749 (8)	0.2885 (12)	0.036 (5)*	
H19A	−0.179 (3)	0.3514 (8)	0.3763 (12)	0.036 (5)*	
H20A	0.035 (3)	0.3861 (7)	0.4993 (10)	0.020 (4)*	
H22A	1.423 (3)	0.5431 (8)	0.8233 (11)	0.028 (4)*	
H23A	1.660 (3)	0.5687 (8)	0.7123 (11)	0.027 (4)*	
H24A	1.547 (3)	0.5514 (7)	0.5628 (11)	0.026 (4)*	
H25A	1.202 (3)	0.5110 (7)	0.5228 (11)	0.024 (4)*	
H26A	0.975 (3)	0.4861 (8)	0.6324 (11)	0.027 (4)*	
H27A	0.695 (3)	0.3575 (8)	0.9527 (12)	0.031 (4)*	
H27B	0.860 (3)	0.3318 (8)	0.8870 (11)	0.029 (4)*	
H27C	0.629 (3)	0.3524 (8)	0.8503 (12)	0.034 (5)*	

### Atomic displacement parameters (Å^2^)

**Table:**

	*U*^11^	*U*^22^	*U*^33^	*U*^12^	*U*^13^	*U*^23^
Cl1	0.0483 (2)	0.01405 (14)	0.02701 (16)	−0.00199 (14)	0.00901 (14)	−0.00136 (10)
O1	0.0177 (5)	0.0229 (4)	0.0256 (4)	0.0039 (4)	−0.0005 (4)	−0.0005 (3)
N1	0.0146 (5)	0.0189 (5)	0.0162 (4)	−0.0007 (4)	0.0013 (4)	0.0001 (3)
C1	0.0140 (6)	0.0171 (5)	0.0155 (5)	−0.0004 (4)	−0.0017 (4)	0.0008 (4)
C2	0.0133 (5)	0.0175 (5)	0.0139 (5)	0.0000 (4)	−0.0003 (4)	−0.0004 (4)
C3	0.0167 (6)	0.0222 (6)	0.0165 (5)	0.0001 (5)	0.0022 (4)	−0.0024 (4)
C4	0.0214 (6)	0.0207 (6)	0.0183 (5)	0.0024 (5)	0.0026 (5)	−0.0038 (4)
C5	0.0255 (7)	0.0148 (5)	0.0188 (5)	0.0002 (5)	0.0010 (5)	−0.0006 (4)
C6	0.0219 (6)	0.0165 (5)	0.0154 (5)	−0.0003 (5)	0.0020 (5)	0.0011 (4)
C7	0.0157 (6)	0.0162 (5)	0.0122 (4)	−0.0003 (4)	−0.0012 (4)	0.0001 (4)
C8	0.0136 (5)	0.0164 (5)	0.0122 (4)	0.0008 (4)	−0.0004 (4)	0.0004 (4)
C9	0.0148 (6)	0.0156 (5)	0.0132 (4)	0.0012 (4)	−0.0008 (4)	−0.0008 (4)
C10	0.0188 (6)	0.0148 (5)	0.0161 (5)	0.0003 (5)	0.0024 (4)	0.0007 (4)
C11	0.0221 (7)	0.0164 (5)	0.0204 (5)	0.0003 (5)	0.0016 (5)	−0.0035 (4)
C12	0.0221 (6)	0.0160 (5)	0.0178 (5)	−0.0007 (5)	0.0023 (5)	−0.0014 (4)
C13	0.0213 (7)	0.0187 (5)	0.0196 (5)	−0.0005 (5)	0.0003 (5)	−0.0034 (4)
C14	0.0207 (6)	0.0165 (5)	0.0180 (5)	−0.0016 (5)	0.0030 (5)	−0.0017 (4)
C15	0.0198 (6)	0.0162 (5)	0.0172 (5)	−0.0032 (5)	0.0015 (4)	0.0013 (4)
C16	0.0242 (7)	0.0198 (6)	0.0225 (6)	−0.0005 (5)	0.0015 (5)	−0.0027 (4)
C17	0.0345 (8)	0.0226 (6)	0.0225 (6)	−0.0035 (6)	0.0007 (6)	−0.0052 (5)
C18	0.0343 (8)	0.0253 (6)	0.0204 (6)	−0.0063 (6)	−0.0054 (6)	−0.0009 (5)
C19	0.0256 (7)	0.0263 (6)	0.0245 (6)	−0.0015 (6)	−0.0046 (5)	0.0020 (5)
C20	0.0234 (7)	0.0207 (6)	0.0205 (5)	0.0000 (5)	0.0006 (5)	0.0007 (4)
C21	0.0160 (6)	0.0142 (5)	0.0149 (5)	0.0018 (4)	0.0017 (4)	0.0008 (4)
C22	0.0188 (6)	0.0231 (6)	0.0183 (5)	0.0002 (5)	0.0000 (5)	0.0014 (4)
C23	0.0160 (6)	0.0248 (6)	0.0265 (6)	−0.0017 (5)	0.0023 (5)	0.0017 (5)
C24	0.0224 (7)	0.0210 (6)	0.0230 (6)	0.0031 (5)	0.0082 (5)	0.0033 (4)
C25	0.0236 (7)	0.0207 (6)	0.0156 (5)	0.0021 (5)	0.0043 (5)	0.0010 (4)
C26	0.0180 (6)	0.0172 (5)	0.0154 (5)	−0.0001 (5)	0.0008 (4)	−0.0008 (4)
C27	0.0207 (7)	0.0177 (5)	0.0211 (5)	−0.0033 (5)	0.0024 (5)	0.0012 (4)

### Geometric parameters (Å, °)

**Table:**

Cl1—C5	1.7405 (13)	C14—H14A	0.984 (16)
O1—C10	1.2253 (16)	C15—C20	1.3981 (19)
N1—C1	1.3175 (15)	C15—C16	1.4037 (18)
N1—C2	1.3681 (15)	C16—C17	1.3890 (18)
C1—C9	1.4322 (17)	C16—H16A	1.001 (18)
C1—C27	1.5066 (17)	C17—C18	1.389 (2)
C2—C7	1.4180 (17)	C17—H17A	1.000 (19)
C2—C3	1.4217 (16)	C18—C19	1.384 (2)
C3—C4	1.3680 (18)	C18—H18A	0.955 (19)
C3—H3A	0.967 (17)	C19—C20	1.3940 (18)
C4—C5	1.4093 (19)	C19—H19A	1.033 (19)
C4—H4A	0.981 (17)	C20—H20A	0.993 (16)
C5—C6	1.3693 (17)	C21—C22	1.3938 (18)
C6—C7	1.4230 (16)	C21—C26	1.4007 (15)
C6—H6A	0.986 (17)	C22—C23	1.3926 (18)
C7—C8	1.4302 (16)	C22—H22A	0.983 (17)
C8—C9	1.3794 (16)	C23—C24	1.3911 (18)
C8—C21	1.4921 (16)	C23—H23A	0.987 (18)
C9—C10	1.5088 (16)	C24—C25	1.389 (2)
C10—C11	1.4635 (17)	C24—H24A	0.965 (18)
C11—C12	1.3445 (19)	C25—C26	1.3897 (17)
C11—H11A	0.994 (17)	C25—H25A	0.981 (16)
C12—C13	1.4451 (16)	C26—H26A	0.967 (18)
C12—H12A	0.976 (17)	C27—H27A	0.984 (19)
C13—C14	1.3417 (19)	C27—H27B	0.985 (18)
C13—H13A	0.967 (18)	C27—H27C	0.982 (19)
C14—C15	1.4666 (16)		
			
C1—N1—C2	118.65 (10)	C20—C15—C16	118.41 (11)
N1—C1—C9	122.41 (11)	C20—C15—C14	118.64 (11)
N1—C1—C27	116.40 (11)	C16—C15—C14	122.94 (12)
C9—C1—C27	121.18 (11)	C17—C16—C15	120.35 (13)
N1—C2—C7	123.01 (11)	C17—C16—H16A	119.7 (10)
N1—C2—C3	117.28 (11)	C15—C16—H16A	119.9 (10)
C7—C2—C3	119.71 (11)	C16—C17—C18	120.45 (13)
C4—C3—C2	120.41 (12)	C16—C17—H17A	118.2 (11)
C4—C3—H3A	122.8 (10)	C18—C17—H17A	121.3 (10)
C2—C3—H3A	116.8 (10)	C19—C18—C17	119.97 (12)
C3—C4—C5	119.18 (11)	C19—C18—H18A	119.1 (11)
C3—C4—H4A	120.0 (10)	C17—C18—H18A	120.8 (11)
C5—C4—H4A	120.8 (10)	C18—C19—C20	119.79 (14)
C6—C5—C4	122.68 (12)	C18—C19—H19A	123.1 (10)
C6—C5—Cl1	119.38 (10)	C20—C19—H19A	117.1 (10)
C4—C5—Cl1	117.93 (9)	C19—C20—C15	121.03 (13)
C5—C6—C7	118.85 (12)	C19—C20—H20A	119.4 (9)
C5—C6—H6A	119.9 (10)	C15—C20—H20A	119.6 (9)
C7—C6—H6A	121.3 (10)	C22—C21—C26	118.92 (11)
C2—C7—C6	119.16 (11)	C22—C21—C8	121.41 (10)
C2—C7—C8	117.69 (10)	C26—C21—C8	119.66 (11)
C6—C7—C8	123.15 (11)	C23—C22—C21	120.53 (11)
C9—C8—C7	118.19 (11)	C23—C22—H22A	122.6 (11)
C9—C8—C21	120.91 (10)	C21—C22—H22A	116.8 (11)
C7—C8—C21	120.88 (10)	C24—C23—C22	120.21 (13)
C8—C9—C1	120.05 (10)	C24—C23—H23A	120.3 (10)
C8—C9—C10	119.50 (11)	C22—C23—H23A	119.5 (10)
C1—C9—C10	120.15 (10)	C25—C24—C23	119.56 (12)
O1—C10—C11	120.69 (11)	C25—C24—H24A	120.7 (10)
O1—C10—C9	118.97 (10)	C23—C24—H24A	119.7 (10)
C11—C10—C9	120.34 (11)	C24—C25—C26	120.43 (11)
C12—C11—C10	124.93 (12)	C24—C25—H25A	119.9 (10)
C12—C11—H11A	120.4 (10)	C26—C25—H25A	119.7 (10)
C10—C11—H11A	114.6 (10)	C25—C26—C21	120.35 (12)
C11—C12—C13	123.17 (12)	C25—C26—H26A	119.2 (10)
C11—C12—H12A	121.2 (10)	C21—C26—H26A	120.4 (10)
C13—C12—H12A	115.7 (10)	C1—C27—H27A	109.8 (10)
C14—C13—C12	122.85 (12)	C1—C27—H27B	114.0 (10)
C14—C13—H13A	121.6 (10)	H27A—C27—H27B	109.3 (14)
C12—C13—H13A	115.5 (10)	C1—C27—H27C	109.3 (11)
C13—C14—C15	126.69 (12)	H27A—C27—H27C	107.8 (15)
C13—C14—H14A	116.9 (9)	H27B—C27—H27C	106.4 (15)
C15—C14—H14A	116.4 (9)		
			
C2—N1—C1—C9	0.42 (17)	C1—C9—C10—O1	−105.62 (14)
C2—N1—C1—C27	179.34 (10)	C8—C9—C10—C11	−111.08 (13)
C1—N1—C2—C7	−0.12 (17)	C1—C9—C10—C11	75.19 (14)
C1—N1—C2—C3	179.31 (11)	O1—C10—C11—C12	−175.74 (12)
N1—C2—C3—C4	−179.28 (11)	C9—C10—C11—C12	3.44 (19)
C7—C2—C3—C4	0.17 (18)	C10—C11—C12—C13	175.68 (12)
C2—C3—C4—C5	−0.14 (19)	C11—C12—C13—C14	−177.89 (13)
C3—C4—C5—C6	0.4 (2)	C12—C13—C14—C15	176.62 (12)
C3—C4—C5—Cl1	−178.59 (10)	C13—C14—C15—C20	−176.86 (13)
C4—C5—C6—C7	−0.74 (19)	C13—C14—C15—C16	2.0 (2)
Cl1—C5—C6—C7	178.29 (9)	C20—C15—C16—C17	−0.23 (19)
N1—C2—C7—C6	178.96 (11)	C14—C15—C16—C17	−179.12 (12)
C3—C2—C7—C6	−0.46 (17)	C15—C16—C17—C18	0.1 (2)
N1—C2—C7—C8	0.03 (17)	C16—C17—C18—C19	0.2 (2)
C3—C2—C7—C8	−179.39 (11)	C17—C18—C19—C20	−0.3 (2)
C5—C6—C7—C2	0.73 (18)	C18—C19—C20—C15	0.1 (2)
C5—C6—C7—C8	179.60 (11)	C16—C15—C20—C19	0.11 (19)
C2—C7—C8—C9	−0.23 (16)	C14—C15—C20—C19	179.05 (12)
C6—C7—C8—C9	−179.12 (11)	C9—C8—C21—C22	−118.31 (13)
C2—C7—C8—C21	178.30 (10)	C7—C8—C21—C22	63.20 (16)
C6—C7—C8—C21	−0.59 (17)	C9—C8—C21—C26	61.54 (15)
C7—C8—C9—C1	0.52 (16)	C7—C8—C21—C26	−116.95 (13)
C21—C8—C9—C1	−178.01 (10)	C26—C21—C22—C23	−0.23 (18)
C7—C8—C9—C10	−173.22 (10)	C8—C21—C22—C23	179.63 (12)
C21—C8—C9—C10	8.26 (16)	C21—C22—C23—C24	−0.5 (2)
N1—C1—C9—C8	−0.64 (17)	C22—C23—C24—C25	0.9 (2)
C27—C1—C9—C8	−179.51 (11)	C23—C24—C25—C26	−0.54 (19)
N1—C1—C9—C10	173.05 (11)	C24—C25—C26—C21	−0.24 (19)
C27—C1—C9—C10	−5.82 (16)	C22—C21—C26—C25	0.62 (18)
C8—C9—C10—O1	68.11 (15)	C8—C21—C26—C25	−179.24 (11)

### Hydrogen-bond geometry (Å, °)

**Table:**

*D*—H···*A*	*D*—H	H···*A*	*D*···*A*	*D*—H···*A*
C3—H3A···N1^i^	0.966 (17)	2.484 (19)	3.3681 (16)	152.1 (14)
C11—H11A···Cl1^ii^	0.994 (17)	2.772 (16)	3.6491 (12)	147.4 (14)
C17—H17A···O1^iii^	1.000 (19)	2.596 (19)	3.4398 (17)	142.1 (14)
C27—H27C···O1^iv^	0.984 (18)	2.517 (19)	3.3892 (16)	147.6 (14)

Symmetry codes: (i) −*x*+1, −*y*+1, −*z*+2; (ii) −*x*+2, *y*−1/2, −*z*+3/2; (iii) *x*−1, −*y*+1/2, *z*−1/2; (iv) *x*−1, *y*, *z*.
